# Prevalence of FH-Causing Variants and Impact on LDL-C Concentration in European, South Asian, and African Ancestry Groups of the UK Biobank—Brief Report

**DOI:** 10.1161/ATVBAHA.123.319438

**Published:** 2023-07-06

**Authors:** Jasmine Gratton, Steve E. Humphries, Marta Futema

**Affiliations:** Institute of Cardiovascular Science, Faculty of Population Health Sciences, University College London, United Kingdom (J.G., S.E.H., M.F.).; Cardiology Research Centre, Molecular and Clinical Sciences Research Institute, St George’s University of London, United Kingdom (M.F.).

**Keywords:** cholesterol, LDL, hyperlipoproteinemia type II, receptors, LDL, sequence analysis, DNA

## Abstract

**Background:**

Familial hypercholesterolemia (FH) is a monogenic disease that causes high low-density lipoprotein cholesterol (LDL-C) and higher risk of premature coronary heart disease. The prevalence of FH-causing variants and their association with LDL-C in non-European populations remains largely unknown. Using DNA diagnosis in a population-based cohort, we aimed to estimate the prevalence of FH across 3 major ancestry groups in the United Kingdom.

**Methods:**

Principal component analysis was used to distinguish genetic ancestry in UK Biobank participants. Whole exome sequencing data were analyzed to provide a genetic diagnosis of FH. LDL-C concentrations were adjusted for statin use.

**Results:**

Principal component analysis distinguished 140 439 European, 4067 South Asian, and 3906 African participants with lipid and whole exome sequencing data. There were significant differences between the 3 groups, including total and LDL-C concentrations, and prevalence and incidence of coronary heart disease. We identified 488, 18, and 15 participants of European, South Asian, and African ancestry carrying a likely pathogenic or pathogenic FH-variant. No statistical difference in the prevalence of an FH-causing variant was observed: 1 out of 288 (95% CI, 1/316–1/264) in European, 1 out of 260 (95% CI, 1/526–1/173) in African, and 1 out of 226 (95% CI, 1/419–1/155) in South Asian. Carriers of an FH-causing variant had significantly higher LDL-C concentration than noncarriers in every ancestry group. There was no difference in median (statin-use adjusted) LDL-C concentration in FH-variant carriers depending on their ancestry background. Self-reported statin use was nonsignificantly highest in FH-variant carriers of South Asian ancestry (55.6%), followed by African (40.0%) and European (33.8%; *P*=0.15).

**Conclusions:**

The prevalence of FH-causing variants in the UK Biobank is similar across the ancestry groups analyzed. Despite overall differences in lipid concentrations, FH-variant carriers across the 3 ancestry groups had similar LDL-C levels. In all ancestry groups, the proportion of FH-variant carriers treated with lipid-lowering therapy should be improved to reduce future risk of premature coronary heart disease.

**Highlights**

- Prevalence of familial hypercholesterolemia–causing variants is similar across European, South Asian, and African genetic ancestry groups of UK Biobank.
- Despite some overall differences in lipid concentrations between the ancestry groups, individuals with familial hypercholesterolemia have similar LDL (low-density lipoprotein)-cholesterol regardless of their ancestry.
- Gaps in lipid-lowering treatment exist across all 3 ancestry groups.

Familial hypercholesterolemia (FH) is a monogenic disease of high low-density lipoprotein cholesterol (LDL-C) concentrations caused by rare variants in the *LDLR*, *APOB*, or *PCSK9* gene.^1^ Carriers of an FH-causing variant are predisposed to a high risk of premature coronary heart disease (CHD) because they are exposed to increased LDL-C concentrations from birth.^2^ Likely pathogenic and pathogenic variants in *LDLR*, *APOB*, or *PCSK9* can be found in 1 in 250 individuals (95% CI, 1:345–1:192), as demonstrated in a recent meta-analysis.^3^ Despite FH being one of the most common Mendelian diseases, it remains highly underdiagnosed worldwide,^1^ and even when diagnosed, it is often inadequately treated.^4^ The majority of available data on the prevalence of FH come from studies conducted on individuals of European ancestry; therefore, significant gaps in the understanding of the disease burden in non-European ancestry groups remain.^5^ A previous study using the predominately male Million Veteran Program data observed differences in FH-variant frequency between individuals of European and African background^6^; however, the authors used a genotyping array for FH-variant identification, which is likely to miss some rare pathogenic variants, especially those unique to non-European populations. A recent systematic review observed differences in FH prevalence between different ethnicities; however, FH diagnosis was mainly based on a mixture of clinical criteria with a limited number being confirmed by genetic testing.^7^ One of the research needs highlighted in the Scientific Statement from the American Heart Association is to determine the prevalence of FH-variants in non-European populations.^8^ Potential differences in FH-variant frequency between ancestry groups are important to consider when evaluating national screening strategies.

In this study, we assessed the prevalence of FH-causing variants in the UK Biobank and compared it between the 3 major ancestry groups available: European, South Asian (SA), and African. We analyzed differences in LDL-C concentration between FH-variant carriers across these 3 groups and compared them with non-FH participants.

## METHODS

Because of the sensitive nature of the data generated for this study, requests to access the raw dataset from qualified researchers trained in human subject confidentiality protocols may be sent to the UK Biobank at https://www.ukbiobank.ac.uk/enable-your-research/apply-for-access.

### UK Biobank Cohort

The UK Biobank longitudinal study recruited half a million participants (aged 40–75 years) from 2006 to 2010.^9^ Various biological (eg, biomarker measures) and nonbiological (eg, lifestyle, family history of disease) measures were recorded, including whole exome sequencing of participants.^10^ The current study was conducted under the approved UK Biobank application 40721.

### Principal Component Analysis

The genetic ancestry of participants was determined using principal component analysis of the genotyping data as previously described in more detail in the supplemental content of the article by Giannakopoulu et al.^11^ In principal component analysis, participants were confirmed to belong to an ancestry group based on whether they clustered around a known reference group using the PC-Air method.^12^

### Whole Exome Sequencing Analysis

The *LDLR*, *APOB*, and *PCSK9* gene regions ±100 kb (genomic positions shown in Table S1) were extracted from the whole exome sequencing data for available participants at the time of study completion ≈200 000). Variants were first filtered based on a minimum read depth of 10, genotype quality of 20, and a minor allele frequency of ≤0.0006 (ie, the frequency of the most common FH variant p.Arg3527Gln in *APOB*).

### Variant Interpretation

Variants that passed the initial quality control were interpreted using the American College of Medical Genetics guidelines.^13^ Variants classified as likely pathogenic or pathogenic in Table S2 were used to provide a genetic diagnosis of FH. Variants of uncertain significance (VUS) were excluded from the main analysis and presented in Table S3. FH-causing variants in the *APOB* and *PCSK9* genes were filtered based on a list of variants with functional assay backing.^14^ The frequency of the likely pathogenic p.Leu167del variant in *APOE* was also analyzed.^15^

### LDL-C Concentration Data

Missing LDL-C data was singly imputed using the R package MICE version 3.10.0.^16^ The LDL-C concentration of participants who reported using statins was adjusted with the correction coefficient 1.43.^17^

### Statistical Methods

All statistical analyses were performed in R version 4.0.2. The nonparametric Kruskal-Wallis rank-sum test was used to compare the median (interquartile range [IQR]) of >2 groups, and the Mann-Whitney *U* test–Wilcoxon Test when comparing 2 groups.

## RESULTS

### Ancestry Groups Characteristics

Using principal component analysis, we identified 140 439 White European, 4067 SA, and 3906 African ancestry individuals with whole exome sequencing data within UK Biobank. Significant differences between the 3 groups were observed (Table S4), with the median age being slightly older in European (58 versus 53 years in SA, and 50 years in African ancestry, *P*<0.001) and having highest total cholesterol and LDL-C concentrations (median [IQR] total cholesterol, 5.68 [4.94–6.45] mmol/L versus 5.32 [4.56–6.03] mmol/L in SA and 5.24 [4.52–5.94] mmol/L in African, *P*<0.001; LDL-C adjusted for statin use, 3.67 [3.14–4.25] mmol/L versus 3.54 [3.02–4.08] mmol/L in SA and 3.36 [2.84–3.95] mmol/L in African, *P*<0.001). Median [IQR] triglyceride concentrations were highest in SA group: 1.67 [1.18–2.40] mmol/L (versus 1.49 [1.05–2.14] mmol/L in European and 1.04 [0.78–1.46] mmol/L in African, *P*<0.001), who also had the highest proportion of prevalent and incident type 2 diabetes and CHD and of prevalent hypertension (Table S4). Median Lp(a) (lipoprotein [a]) values were significantly higher in the African ancestry group, followed by the SA and European groups (overall *P*<0.001; Table S4). The lowest proportion of prevalent and incident CHD was observed in the African ancestry group (Table S4).

### Frequency of FH-Causing Variants

FH-causing variants, classified as pathogenic or likely pathogenic (Table S2), were found in 488 European, 18 SA, and 15 African ancestry participants. These equated to an FH-causing variant prevalence of 1:288 (95% CI, 1:315–1:263), 1:226 (95% CI, 1:381–1:143), and 1:260 (95% CI, 1:465–1:158), respectively, which was not statistically different between the ancestry groups (*P*=0.57; Table 1). The majority of FH-causing variants were seen in the *LDLR* gene, with *APOB* variants (p.Arg3527Gln and p.Arg3527Trp) accounting for 21%, 20%, and 6% FH causes in those of European, African, and SA ancestry, respectively. No pathogenic or likely pathogenic FH-causing variants were seen in *PCSK9* in any of the ancestry groups, while the likely pathogenic variant in *APOE* (p.Leu167del) was found only in those of European ancestry. The highest proportion of VUS was found in European (n=661; 1 in 212), followed by African (n=10; 1 in 391) and SA (n=13; 1 in 313) ancestry groups (Table S3), with no significant difference in prevalence between groups (*P*=0.06).

**Table 1.**
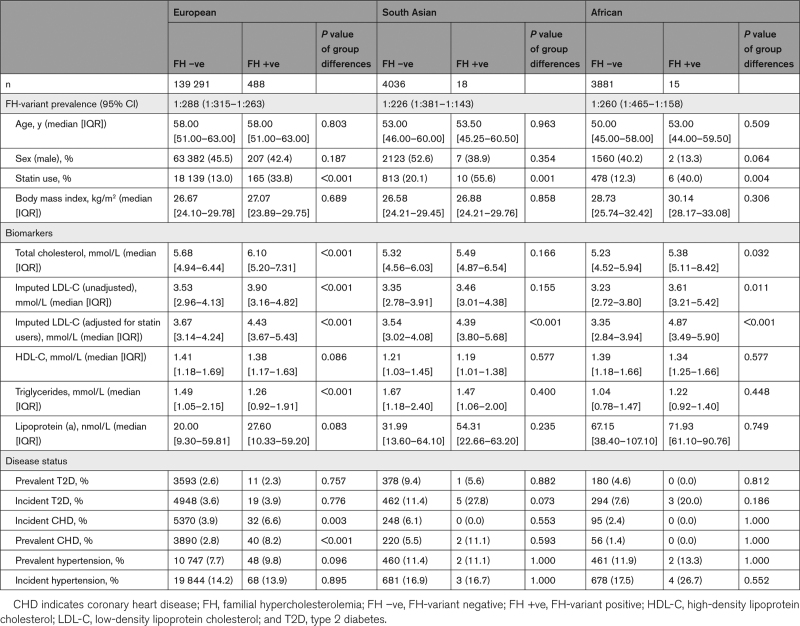
FH-Variant Prevalence and Baseline Characteristics of European, South Asian, and African Ancestry Groups Stratified by Variant Status

### FH-Causing Variant and LDL-C Concentrations

Individuals with African ancestry and an FH-causing variant had the highest median [IQR] adjusted LDL-C concentration (4.87 [3.49–5.90] mmol/L), followed by European (4.43 mmol/L [3.67–5.43 mmol/L]), and SA (4.39 mmol/L [3.80–5.68 mmol/L]; Table 1); however, the differences were not statistically significantly (*P* value of ancestry group differences for FH-variant carriers only=0.67; Table 1). African ancestry individuals without an FH variant had the lowest median [IQR] adjusted LDL-C concentration (3.35 [2.84–3.94] mmol/L; Table 1), which differed significantly between the groups (*P* value of ancestry group differences for non-FH–variant carriers <2.2×10^–16^). Carriers of an FH-causing variant had significantly higher LDL-C concentration than noncarriers in all ancestry groups (Figure). The median [IQR] LDL-C concentration (adjusted for statin use) in participants with a VUS was intermediate between the FH-positive and FH-negative groups in European (3.91 [3.40, 4.55]mmol/L; *P*<0.001) and African (3.83 [3.13, 4.78] mmol/L; *P*<0.001), and lowest in SA (2.91 [2.22, 3.80] mmol/L; *P*<0.001).

**Figure.**
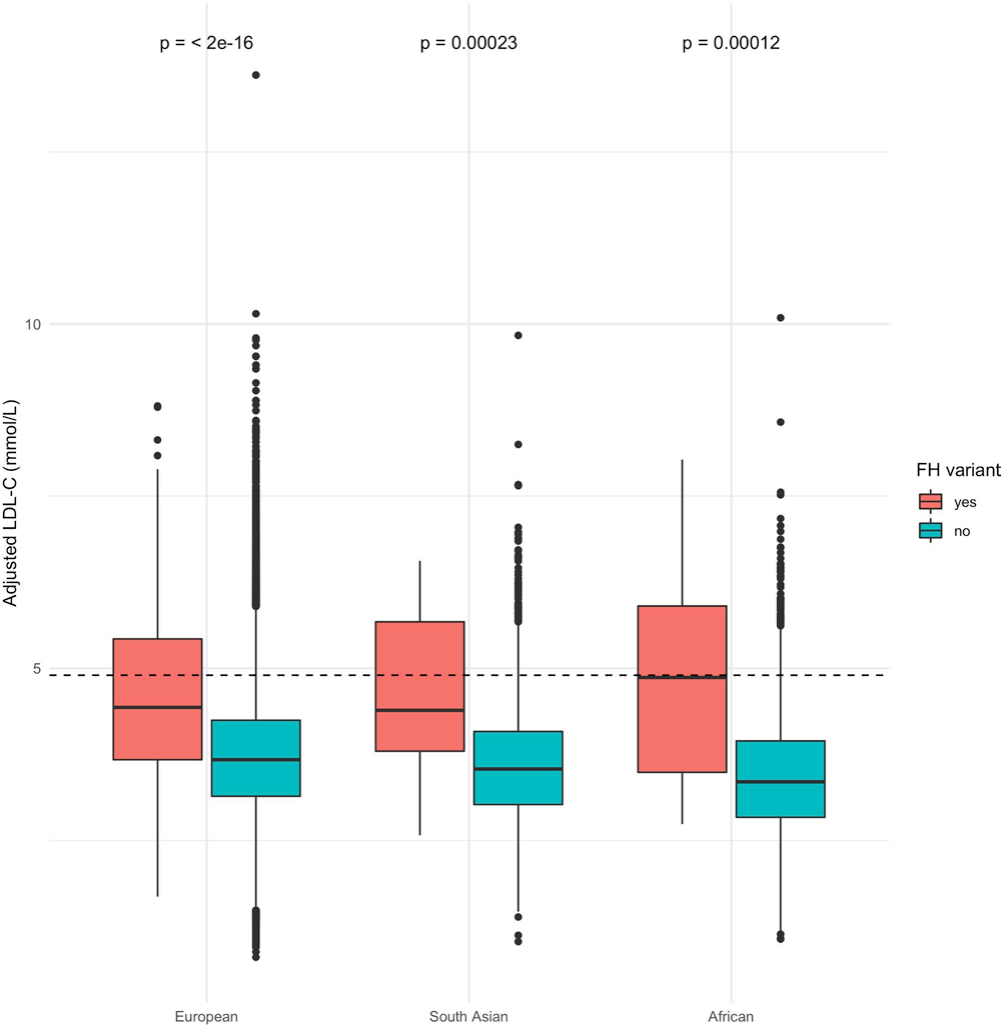
**Low-density lipoprotein cholesterol (LDL-C) concentration in familial hypercholesterolemia (FH) variant carriers and noncarriers in European, South Asian, and African ancestry groups of the UK Biobank.** LDL-C concentration was adjusted for statin use. FH-variant carriers are represented in red, and noncarriers in blue. *P* value differences of LDL-C concentration between carriers and noncarriers are indicated for each ancestry group.

The proportion of FH-positive individuals with LDL-C concentration above the Simon Broome FH diagnostic threshold of 4.9 mmol/L was not statistically different between the ancestries: 46.7% (95% CI, 24.8%–70.0%) in African, 37.7% (95% CI, 33.5%–42.1%) in European, and 33.3% (95% CI, 16.3%–56.3%) in SA (Table S5).^18^ The highest detection rate and the lowest false positive rate when using the diagnostic LDL-C cutoff of 4.9 mmol/L was achieved in African ancestry group (detection rate, 46.7% [95% CI, 24.8%–70.0%]; false positive rate, 5.7% [95% CI, 5.0%–6.4%]) albeit overlapping CIs between groups (Table S5).

### Self-Reported Statin Use

Overall, self-reported statin use was highest in SA (20.3%), with 55.6% of SA FH-variant carriers being treated (Table S6). Among European and African ancestry individuals, 13.1% and 12.5% have reported using statins, with 33.8% and 40.0% of FH-variant carriers being treated, respectively. The differences in treatment use between FH-variant carriers of these ancestry groups were not statistically significant according to the Kruskal-Wallis rank sum test (*P*=0.15) and the pairwise Wilcoxon rank sum test (*P*=0.17 between European-SA, *P*=0.78 between European-African, *P*=0.78 between African-SA).

## DISCUSSION

Although mean LDL-C concentration between European, SA, and African ancestry participants of the UK Biobank was significantly different, the frequency of FH-causing variants was not. Racial and ethnic minority groups are underrepresented in FH registries and cardiovascular clinical trials,^5,19^ and our results suggest that it is not due to differences in FH-variant frequency in these populations. We observed that FH-variant carriers have significantly higher mean LDL-C concentration than noncarriers, regardless of their ancestry. However, we found that in all 3 ancestry groups studied, FH-positive individuals were undertreated, therefore not benefiting from lipid-lowering treatment and reduction in CHD risk. In the UK National Health System, lipid-lowering therapy is affordable and readily available, but in countries where medication is more expensive this gap in treatment may be even greater between ancestry groups. We further found that median plasma triglyceride concentrations and the incidence and prevalence of type 2 diabetes and prevalence of hypertension were highest in SA ancestry participants, which, as noted in previous studies,^20,21^ probably contribute to the higher rates of incident and prevalent CHD in this group. The underlying causes for these higher rates are unclear, but is likely to be a combination of both socioeconomic and lifestyle factors, and genetic background. By contrast, and again as noted previously,^20,22^ the prevalence and incidence of cardiovascular disease was lower in those of African origin, although this effect was largely explained by sociodemographic, lifestyle, environmental, and clinical factors.^20^ However, overall, as more efforts are needed to improve FH diagnosis, our findings suggest that FH-variant carriers in all 3 ancestry groups have similar mean LDL-C concentration and that the commonly used FH diagnostic threshold of LDL-C>4.9 mmol/L might perform similarly at detecting affected FH individuals.

As expected, in all 3 ancestry groups, over 77% (80% in African, 94% in SA) of the identified pathogenic/likely pathogenic variants were in the *LDLR* gene, with 91 different variants found in the European, 11 in the SA, and 9 in the African ancestry groups (Table S2). The FH-variant spectrum in SA was different, with 9 out of the 11 *LDLR* variants being unique to SA (the remaining 2 were also found in European individuals). However, in African ancestry group only 2 *LDLR* variants were unique to African, with 7 also found in European. The *APOB* gene 2 previously reported pathogenic variants were identified ^23,24^ while only VUS were identified in the gene for *PCSK9*, and with the *APOE* likely pathogenic variant seen only in the European ancestry group. For the European and African ancestry groups, VUS carriers had intermediate median LDL-C concentration (ie, higher than non-FH but lower than FH-variant carriers), which suggests that some of the VUS are likely to be pathogenic. If this is the case, the prevalence of FH in these ancestry groups would be higher than estimated in this study. These data support the view that a next-generation sequencing approach including all 4 genes is required to provide a comprehensive genetic diagnostic test for FH.^1,5^ A possible limitation to our analyses is that the variant classification, especially VUS, may change in the future as more evidence is gathered and curated by expert panels such as the FH ClinGen consortium.^25^ Such changes may have a slight effect on the FH prevalence, detection rate, and false positive rate values found in our study.

Although the UK Biobank study provides a large dataset for studying FH in different ancestry groups, limitations include the relatively smaller number of non-European ancestry participants. Other study limitations leading to potential inaccuracies of the results are the use of the self-reported statin treatment data field and the adjustment of LDL-C concentration in these individuals. The adjustment we have adopted might result in an overestimation or underestimation of untreated LDL-C concentration, with greater potential bias in the smaller FH-positive African and SA groups, as the true effect will be statin type and dose-dependent as shown previously.^26^ However, this will not influence the FH prevalence estimates obtained using whole exome sequencing. Regardless of the limitations, this study provides important information on the genetically confirmed prevalence of FH in individuals of African and SA ancestries.

## ARTICLE INFORMATION

### Acknowledgments

This research has been conducted using data from UK Biobank (application number 40721), a major biomedical database. The authors are grateful to UK Biobank participants. UK Biobank was established by the Wellcome Trust medical charity, Medical Research Council, Department of Health, Scottish Government, and the Northwest Regional Development Agency. It has also had funding from the Welsh Assembly Government and the British Heart Foundation.

### Sources of Funding

J. Gratton was supported by the British Heart Foundation studentship FS/17/70/33482. S.E. Humphries and M. Futema received additional support from the National Institute for Health Research University College London Hospitals Biomedical Research Center. S.E Humphries and M. Futema were supported by a grant from the British Heart Foundation (BHF grant PG 08/008).

### Disclosures

None.

### Supplemental Material

Supplemental Methods

Table S1–S6

## Supplementary Material


