# Supplementary material for: Prevalence of FH-Causing Variants and Impact on LDL-C Concentration in European, South Asian, and African Ancestry Groups of the UK Biobank—Brief Report
Source: Arterioscler Thromb Vasc Biol. 2023 Jul 6;43(9):1737–42. doi: 10.1161/ATVBAHA.123.319438 (PMC10443626; doi:10.1161/ATVBAHA.123.319438)
Supplement: Supplementary file 1 [file atv-43-1737-s001.pdf]

**SUPPLEMENTARY MATERIAL**

# Prevalence of familial hypercholesterolaemia (FH)-causing variants and impact on LDL-C concentration in European, South Asian, and African ancestry groups of the UK Biobank

Jasmine Gratton[1], Steve E Humphries[1], Marta Futema[1,2]

[1]Institute of Cardiovascular Science, Faculty of Population Health Sciences, University College London, London, United Kingdom

[2]Cardiology Research Centre, Molecular and Clinical Sciences Research Institute, St George's University of London, London, United Kingdom

**Expanded Methods:**

**Disease definition**

CHD was defined using ICD-10 codes I21, I22, I23, I24, I25.0, I25.1, I25.2, I25.3, I25.5, 125.6, I25.8, 125.9, I48, I50, I11.0, I13.0, I13.2, I32.2, I61, I63 and the OPCS-4 codes K40, K41, K42, K43, K44.1, K44.8, K44.9, K45.1, K45.2, K45.3, K45.4, K45.5, K45.8, K45.9, K46.1, K46.2, K46.3, K46.4, K46.8, K46.9, K47.1, K49, K50, K75, K52.1, K57.1, K57.5, K62.1, K62.2, K62.3, K62.4, K62.5, X50.1, X50.2. Type 2 diabetes was defined using the ICD10 code E11. Hypertension was defined as a composite of self-reported antihypertensive medication use, self-reported hypertension, and the ICD-10 code I10.

**Supplementary Table S1. Genetic coordinates of the *LDLR, APOE, APOB* and *PCSK9* genes.** The genetic coordinates used to extract the FH-causing genes from the whole exome sequencing data are mapped to GRCh38.

| Gene name | Chromosome number | Start coordinate | End coordinate |
|---|---|---|---|
| *LDLR* | 19 | 11,089,262 | 11,133,820 |
| *APOB* | 2 | 21,001,429 | 21,044,073 |
| *APOE* | 19 | 44,905,791 | 44,909,393 |
| *PCSK9* | 1 | 55,039,347 | 55,064,852 |

**Supplementary Table S2. Pathogenic and likely pathogenic FH-variants identified. A.** in European ancestry, **B.** in South Asian ancestry, **C.** in African ancestry. Genetic coordinates are mapped to GRCh38. The asterisk symbol (*) next to a variant in tables B and C indicates variants that are unique to the ancestry group.

**A. European ancestry**

| Gene | Chromosome | Position | Reference allele | Alternate allele | Nucleotide change | Protein | Number of carriers | UKB frequency (1/n) |
|---|---|---|---|---|---|---|---|---|
| *APOB* | 2 | 21006289 | G | A | c.10579C>T | p.Arg3527Trp | 2 | 70,220 |
| | | 21006288 | C | T | c.10580G>A | p.Arg3527Gln | 99 | 1,419 |
| *APOE* | 19 | 44908791 | GCTC | G | c.499_501del | p.Leu167del | 13 | 10,803 |
| *LDLR* | 19 | 11100236 | C | G | c.81C>G | p.Cys27Trp | 1 | 140,439 |
| | | 11100291 | T | G | c.136T>G | p.Cys46Gly | 1 | 140,439 |
| | | 11100294 | G | A | c.139G>A | p.Asp47Asn | 5 | 28,088 |
| | | 11102705 | C | T | c.232C>T | p.Arg78Cys | 13 | 10,803 |
| | | 11102714 | C | T | c.241C>T | p.Arg81Cys | 2 | 70,220 |
| | | 11102732 | T | G | c.259T>G | p.Trp87Gly | 6 | 23,407 |
| | | 11102741 | G | A | c.268G>A | p.Asp90Asn | 5 | 28,088 |
| | | 11102765 | G | A | c.292G>A | p.Gly98Ser | 10 | 14,044 |
| | | 11102774 | G | A | c.301G>A | p.Glu101Lys | 12 | 11,703 |
| | | 11102787 | G | A | c.313+1G>A | . | 5 | 28,088 |
| | | 11102787 | G | C | c.313+1G>C | . | 1 | 140,439 |
| | | 11102787 | G | GT | c.313+2dup | . | 2 | 70,220 |
| | | 11105249 | C | T | c.343C>T | p.Arg115Cys | 2 | 70,220 |
| | | 11105268 | G | T | c.362G>T | p.Cys121Phe | 2 | 70,220 |

| | | | | | | |
|---|---|---|---|---|---|---|
| 11105324 | G | A | c.418G>A | p.Glu140Lys | 1 | 140,439 |
| 11105339 | GTGCTCACCTGTGGTCCCGCCAGC | G | c.435_457del | p.Leu146ProfsTer26 | 1 | 140,439 |
| 11105407 | C | A | c.501C>A | p.Cys167Ter | 2 | 70,220 |
| 11105408 | G | A | c.502G>A | p.Asp168Asn | 14 | 10,031 |
| 11105415 | AC | A | c.513del | p.Asp172ThrfsTer34 | 1 | 140,439 |
| 11105448 | C | G | c.542C>G | p.Pro181Arg | 2 | 70,220 |
| 11105549 | C | T | c.643C>T | p.Arg215Cys | 4 | 35,110 |
| 11105567 | G | A | c.661G>A | p.Asp221Asn | 2 | 70,220 |
| 11105568 | A | G | c.662A>G | p.Asp221Gly | 5 | 28,088 |
| 11105585 | GAC | G | c.680_681del | p.Asp227GlyfsTer12 | 4 | 35,110 |
| 11105585 | GAC | GAG | c.681delinsG | p.Asp227Glu | 2 | 70,220 |
| 11105588 | G | T | c.682G>T | p.Glu228Ter | 2 | 70,220 |
| 11105589 | AG | A | c.685del | p.Glu229LysfsTer36 | 1 | 140,439 |
| 11106579 | C | T | c.709C>T | p.Arg237Cys | 1 | 140,439 |
| 11106588 | G | A | c.718G>A | p.Glu240Lys | 20 | 7,022 |
| 11106592 | T | C | c.722T>C | p.Phe241Ser | 1 | 140,439 |
| 11106631 | A | C | c.761A>C | p.Gln254Pro | 1 | 140,439 |
| 11107432 | C | A | c.858C>A | p.Ser286Arg | 1 | 140,439 |
| 11107433 | G | A | c.859G>A | p.Gly287Ser | 4 | 35,110 |
| 11107436 | G | A | c.862G>A | p.Glu288Lys | 1 | 140,439 |
| 11107461 | G | A | c.887G>A | p.Cys296Tyr | 1 | 140,439 |
| 11107481 | C | T | c.907C>T | p.Arg303Trp | 2 | 70,220 |
| 11107486 | C | G | c.912C>G | p.Asp304Glu | 4 | 35,110 |

| | | | | | | |
|---|---|---|---|---|---|---|
| 11107512 | G | A | c.938G>A | p.Cys313Tyr | 2 | 70,220 |
| 11110660 | G | A | c.949G>A | p.Glu317Lys | 35 | 4,013 |
| 11110678 | G | A | c.967G>A | p.Gly323Ser | 1 | 140,439 |
| 11110714 | G | A | c.1003G>A | p.Gly335Ser | 3 | 46,813 |
| 11110738 | G | A | c.1027G>A | p.Gly343Ser | 8 | 17,555 |
| 11110759 | C | T | c.1048C>T | p.Arg350Ter | 4 | 35,110 |
| 11110760 | G | C | c.1049G>C | p.Arg350Pro | 4 | 35,110 |
| 11111571 | G | A | c.1118G>A | p.Gly373Asp | 1 | 140,439 |
| 11111619 | C | T | c.1166C>T | p.Thr389Met | 8 | 17,555 |
| 11113286 | G | A | c.1195G>A | p.Ala399Thr | 1 | 140,439 |
| 11113287 | C | A | c.1196C>A | p.Ala399Asp | 1 | 140,439 |
| 11113292 | CTCTTC | CTCT | c.1205_1206del | p.Phe403HisfsTer37 | 1 | 140,439 |
| 11113307 | C | T | c.1216C>T | p.Arg406Trp | 5 | 28,088 |
| 11113308 | G | A | c.1217G>A | p.Arg406Gln | 4 | 35,110 |
| 11113313 | G | A | c.1222G>A | p.Glu408Lys | 1 | 140,439 |
| 11113322 | A | G | c.1231A>G | p.Lys411Glu | 1 | 140,439 |
| 11113329 | C | T | c.1238C>T | p.Thr413Met | 14 | 10,031 |
| 11113337 | C | T | c.1246C>T | p.Arg416Trp | 2 | 70,220 |
| 11113419 | G | C | c.1328G>C | p.Trp443Ser | 1 | 140,439 |
| 11113426 | C | G | c.1335C>G | p.Asp445Glu | 5 | 28,088 |
| 11113554 | CA | C | c.1379del | p.His460ProfsTer47 | 1 | 140,439 |
| 11113590 | G | T | c.1414G>T | p.Asp472Tyr | 6 | 23,407 |
| 11113608 | G | A | c.1432G>A | p.Gly478Arg | 2 | 70,220 |
| 11113612 | T | C | c.1436T>C | p.Leu479Pro | 2 | 70,220 |

| | | | | | | |
|---|---|---|---|---|---|---|
| 11113620 | G | A | c.1444G>A | p.Asp482Asn | 29 | 4,843 |
| 11113650 | G | A | c.1474G>A | p.Asp492Asn | 1 | 140,439 |
| 11113678 | C | T | c.1502C>T | p.Ala501Val | 5 | 28,088 |
| 11113705 | C | T | c.1529C>T | p.Thr510Met | 3 | 46,813 |
| 11113743 | G | A | c.1567G>A | p.Val523Met | 1 | 140,439 |
| 11116095 | T | G | c.1588T>G | p.Phe530Val | 10 | 14,044 |
| 11116125 | G | A | c.1618G>A | p.Ala540Thr | 2 | 70,220 |
| 11116141 | G | A | c.1634G>A | p.Gly545Glu | 1 | 140,439 |
| 11116198 | A | G | c.1691A>G | p.Asn564Ser | 2 | 70,220 |
| 11116873 | C | T | c.1720C>T | p.Arg574Cys | 2 | 70,220 |
| 11116898 | T | C | c.1745T>C | p.Leu582Pro | 1 | 140,439 |
| 11116918 | G | A | c.1765G>A | p.Asp589Asn | 1 | 140,439 |
| 11116928 | G | A | c.1775G>A | p.Gly592Glu | 1 | 140,439 |
| 11116936 | C | T | c.1783C>T | p.Arg595Trp | 6 | 23,407 |
| 11116937 | G | A | c.1784G>A | p.Arg595Gln | 2 | 70,220 |
| 11116976 | C | G | c.1823C>G | p.Pro608Arg | 1 | 140,439 |
| 11120091 | G | A | c.1846-1G>A | . | 1 | 140,439 |
| 11120106 | G | T | c.1860G>T | p.Trp620Cys | 1 | 140,439 |
| 11120110 | GAT | G | c.1867_1868del | p.Ile623HisfsTer21 | 1 | 140,439 |
| 11120143 | C | T | c.1897C>T | p.Arg633Cys | 9 | 15,604 |
| 11120144 | G | A | c.1898G>A | p.Arg633His | 1 | 140,439 |
| 11120152 | G | A | c.1906G>A | p.Gly636Ser | 3 | 46,813 |
| 11120212 | C | A | c.1966C>A | p.His656Asn | 8 | 17,555 |
| 11120370 | G | A | c.1988G>A | p.Gly663Glu | 1 | 140,439 |
| 11120408 | G | A | c.2026G>A | p.Gly676Ser | 5 | 28,088 |

| | | | | | | | | |
|---|---|---|---|---|---|---|---|---|
| | | 11120436 | C | T | c.2054C>T | p.Pro685Leu | 12 | 11,703 |
| | | 11120441 | A | T | c.2059A>T | p.Ile687Phe | 5 | 28,088 |
| | | 11120442 | T | TC | c.2061dup | p.Asn688GlnfsTer29 | 1 | 140,439 |
| | | 11123200 | G | T | c.2167G>T | p.Glu723Ter | 1 | 140,439 |
| | | 11128027 | C | CA | c.2332dup | p.Arg778LysfsTer4 | 1 | 140,439 |

**B. South Asian ancestry**

| Gene | Chromosome | Position | Reference allele | Alternate allele | Nucleotide change | Protein | Number of carriers | UKB frequency (1/n) |
|---|---|---|---|---|---|---|---|---|
| *APOB* | 2 | 21006289 | G | A | c.10579C>T | p.Arg3527Trp | 1 | 4067 |
| *LDLR* | 19 | 11105579 | A | T | c.673A>T | p.Lys225Ter* | 1 | 4067 |
| | | 11110690 | C | T | c.979C>T | p.His327Tyr* | 6 | 2034 |
| | | 11110714 | G | A | c.1003G>A | p.Gly335Ser | 1 | 4067 |
| | | 11111514 | A | G | c.1061A>G | p.Asp354Gly* | 1 | 4067 |
| | | 11113343 | G | A | c.1252G>A | p.Glu418Lys* | 1 | 4067 |
| | | 11113348 | C | G | c.1257C>G | p.Tyr419Ter* | 1 | 4067 |
| | | 11116141 | G | A | c.1634G>A | p.Gly545Glu | 2 | 2034 |
| | | 11116900 | C | T | c.1747C>T | p.His583Tyr* | 1 | 4067 |
| | | 11116901 | A | G | c.1748A>G | p.His583Arg* | 1 | 4067 |
| | | 11120101 | T | C | c.1855T>C | p.Phe619Leu* | 1 | 4067 |
| | | 11120478 | C | T | c.2096C>T | p.Pro699Leu* | 1 | 4067 |

**C. African ancestry**

| Gene | Chromosome | Position | Reference allele | Alternate allele | Nucleotide change | Protein | Number of carriers | UKB frequency (1/n) |
|---|---|---|---|---|---|---|---|---|
| *APOB* | 2 | 21006288 | C | T | c.10580G>A | p.Arg3527Gln | 3 | 1302 |
| *LDLR* | 19 | 11107436 | G | A | c.862G>A | p.Glu288Lys | 1 | 3906 |
| | | 11107481 | C | T | c.907C>T | p.Arg303Trp | 2 | 1953 |
| | | 11107484 | G | A | c.910G>A | p.Asp304Asn* | 1 | 3906 |
| | | 11110678 | G | A | c.967G>A | p.Gly323Ser | 3 | 1302 |

| | | | | | | |
|---|---|---|---|---|---|---|
| 11111571 | G | A | c.1118G>A | p.Gly373Asp | 1 | 3906 |
| 11111619 | C | T | c.1166C>T | p.Thr389Met | 1 | 3906 |
| 11113678 | C | T | c.1502C>T | p.Ala501Val | 1 | 3906 |
| 11116114 | G | A | c.1607G>A | p.Trp536Ter* | 1 | 3906 |
| 11120144 | G | A | c.1898G>A | p.Arg633His | 1 | 3906 |

**Supplementary Table S3. Variants of uncertain significance (VUS) excluded from the main analysis. A.** in European ancestry, **B.** in South Asian ancestry, **C.** in African ancestry. Genetic coordinates are mapped to GRCh38.

## A. European ancestry

| Gene | Chromosome | Position | Reference allele | Alternate allele | Nucleotide change | Protein | Number of carriers | UKB frequency (1/n) |
|---|---|---|---|---|---|---|---|---|
| *APOB* | 2 | 21001939 | ACTG | A | c.13480_13482delCAG | p.Gln4494del | 132 | 1064 |
| | | 21006196 | C | T | c.10672C>T | p.Arg3558Cys | 299 | 470 |
| | | 21006239 | C | G | c.10629C>G | p.Asn3543Lys | 3 | 46813 |
| | | 21006349 | C | T | c.10519C>T | p.Arg3507Trp | 1 | 140438 |
| | | 21015387 | G | C | c.3491G>C | p.Arg1164Thr | 1 | 140438 |
| *PCSK9* | 1 | 55044021 | A | G | c.386A>G | p.Asp129Gly | 2 | 70219 |
| | | 55052698 | G | A | c.706G>A | p.Gly236Ser | 4 | 35110 |
| | | 55058543 | C | G | c.1399C>G | p.Pro467Ala | 3 | 46813 |
| *LDLR* | 19 | 11100261 | G | C | c.106G>C | p.Asp36His | 1 | 140438 |
| | | 11100322 | C | T | c.167C>T | p.Ser56Phe | 1 | 140438 |
| | | 11100328 | A | T | c.173A>T | p.Glu58Val | 2 | 70219 |
| | | 11100340 | C | T | c.185C>T | p.Thr62Met | 10 | 14044 |
| | | 11102720 | A | T | c.247A>T | p.Ile83Phe | 1 | 140438 |
| | | 11105262 | G | C | c.356G>C | p.Gly119Ala | 1 | 140438 |
| | | 11105337 | C | T | c.431C>T | p.Pro144Leu | 1 | 140438 |
| | | 11105379 | C | T | c.473C>T | p.Ser158Phe | 1 | 140438 |
| | | 11105414 | G | A | c.508G>A | p.Asp170Asn | 22 | 6384 |
| | | 11105415 | AC | GC | c.509delinsG | p.Asp170Gly | 1 | 140438 |
| | | 11106580 | G | A | c.710G>A | p.Arg237His | 10 | 14044 |
| | | 11106593 | C | A | c.723C>A | p.Phe241Leu | 3 | 46813 |
| | | 11106601 | C | G | c.731C>G | p.Ser244Cys | 1 | 140438 |
| | | 11106639 | C | T | c.769C>T | p.Arg257Trp | 2 | 70219 |
| | | 11107472 | A | G | c.898A>G | p.Arg300Gly | 1 | 140438 |
| | | 11111538 | A | C | c.1085A>C | p.Asp362Ala | 60 | 2341 |
| | | 11111558 | G | A | c.1105G>A | p.Val369Met | 3 | 46813 |
| | | 11111609 | G | T | c.1156G>T | p.Asp386Tyr | 5 | 28088 |
| | | 11113278 | G | T | c.1187G>T | p.Gly396Val | 1 | 140438 |
| | | 11113287 | C | T | c.1196C>T | p.Ala399Val | 1 | 140438 |
| | | 11113292 | CTCTTC | CTCTTG | c.1206delinsG | p.Phe402Leu | 1 | 140438 |
| | | 11113362 | C | T | c.1271C>T | p.Pro424Leu | 3 | 46813 |
| | | 11113374 | A | C | c.1283A>C | p.Asn428Thr | 1 | 140438 |

| | | | | | | |
|---|---|---|---|---|---|---|
| 11113409 | A | G | c.1318A>G | p.Arg440Gly | 4 | 35110 |
| 11113561 | TCTCTTCCTA | TCTCTTACTA | c.1391delinsA | p.Ser464Tyr | 2 | 70219 |
| 11113625 | G | T | c.1449G>T | p.Trp483Cys | 1 | 140438 |
| 11113751 | T | G | c.1575T>G | p.Asp525Glu | 6 | 23406 |
| 11113762 | G | T | c.1586G>T | p.Gly529Val | 1 | 140438 |
| 11116101 | T | C | c.1594T>C | p.Tyr532His | 1 | 140438 |
| 11116132 | T | A | c.1625T>A | p.Ile542Asn | 1 | 140438 |
| 11116205 | C | G | c.1698C>G | p.Ile566Met | 1 | 140438 |
| 11116885 | G | A | c.1732G>A | p.Val578Ile | 2 | 70219 |
| 11116914 | C | G | c.1761C>G | p.Ser587Arg | 4 | 35110 |
| 11116949 | T | C | c.1796T>C | p.Leu599Ser | 4 | 35110 |
| 11116970 | C | A | c.1817C>A | p.Ala606Asp | 14 | 10031 |
| 11120454 | C | T | c.2072C>T | p.Ser691Leu | 4 | 35110 |
| 11120484 | G | T | c.2102G>T | p.Gly701Val | 1 | 140438 |
| 11120507 | A | G | c.2125A>G | p.Arg709Gly | 1 | 140438 |
| 11123315 | C | T | c.2282C>T | p.Thr761Met | 11 | 12767 |
| 11128062 | C | A | c.2366C>A | p.Ala789Asp | 1 | 140438 |
| 11129553 | G | C | c.2430G>C | p.Trp810Cys | 1 | 140438 |
| 11129573 | A | T | c.2450A>T | p.Asn817Ile | 1 | 140438 |
| 11129582 | G | A | c.2459G>A | p.Ser820Asn | 1 | 140438 |
| 11129633 | A | G | c.2510A>G | p.His837Arg | 18 | 7802 |
| 11129653 | G | A | c.2530G>A | p.Gly844Ser | 1 | 140438 |
| 11131299 | G | C | c.2566G>C | p.Glu856Gln | 1 | 140438 |

## B. South Asian ancestry

| Gene | Chromosome | Position | Reference allele | Alternate allele | Nucleotide change | Protein | Number of carriers | UKB frequency (1/n) |
|---|---|---|---|---|---|---|---|---|
| *APOB* | 2 | 21006289 | G | A | c.11477C>T | Thr3826Met | 4 | 1017 |
| *LDLR* | 19 | 11110710 | G | C | c.999G>C | p.Lys333Asn | 1 | 4067 |
| | | 11113542 | C | T | c.1366C>T | p.Leu456Phe | 1 | 4067 |
| | | 11129527 | C | T | c.2404C>T | p.Leu802Phe | 1 | 4067 |
| | | 11129635 | A | G | c.2512A>G | p.Ile838Val | 1 | 4067 |
| *PCSK9* | 1 | 55052698 | G | A | c.706G>A | p.Gly236Ser | 5 | 813 |

## C. African ancestry

| Gene | Chromosome | Position | Reference allele | Alternate allele | Nucleotide change | Protein | Number of carriers | UKB frequency (1/n) |
|---|---|---|---|---|---|---|---|---|
| *APOB* | 2 | 21005391 | C | T | c.11477C>T | p.Thr3826Met | 3 | 1302 |
| | | 21006196 | C | T | c.10672C>T | p.Arg3558Cys | 4 | 977 |
| *LDLR* | 19 | 11106630 | C | G | c.760C>G | p.Gln254Glu | 1 | 3906 |
| | | 11116095 | T | G | c.1588T>G | p.Phe530Val | 1 | 3906 |
| | | 11128037 | G | GAGA | c.2347_2349dup | p.Lys783dup | 1 | 3906 |

**Supplementary Table S4. Baseline characteristics of European, South Asian, and African ancestry groups.** P-value of group differences was calculated using the Kruskal-Wallis Rank Sum Test. Variants of uncertain significance (VUS) were not excluded from this table.

| | **European** | **South Asian** | **African** | **p-value of group differences** | **Missing (%)** |
|---|---|---|---|---|---|
| n | 140439 | 4067 | 3906 | | |
| Age (median [IQR]) | 58.00 [51.00, 63.00] | 53.00 [46.00, 60.00] | 50.00 [45.00, 58.00] | <0.001 | 0.0 |
| Sex (male) (%) | 63878 (45.5) | 2139 (52.6) | 1568 (40.1) | <0.001 | 0.0 |
| Statin use (%) | 18405 (13.1) | 826 (20.3) | 488 (12.5) | <0.001 | 0.0 |
| Body mass index, kg/m2 (median [IQR]) | 26.67 [24.10, 29.78] | 26.57 [24.20, 29.44] | 28.72 [25.75, 32.41] | <0.001 | 0.4 |
| **Biomarkers** | | | | | |
| Total cholesterol, mmol/L (median [IQR]) | 5.68 [4.94, 6.45] | 5.32 [4.56, 6.03] | 5.24 [4.52, 5.94] | <0.001 | 4.9 |
| Imputed LDL-C (unadjusted), mmol/L (median [IQR]) | 3.53 [2.96, 4.13] | 3.36 [2.78, 3.91] | 3.24 [2.72, 3.81] | <0.001 | 0.0 |
| Imputed LDL-C (adjusted for statin users), mmol/L (median [IQR]) | 3.67 [3.14, 4.25] | 3.54 [3.02, 4.08] | 3.36 [2.84, 3.95] | <0.001 | 0.0 |
| HDL-C, mmol/L (median [IQR]) | 1.41 [1.18, 1.69] | 1.21 [1.03, 1.45] | 1.39 [1.18, 1.66] | <0.001 | 12.5 |
| Triglycerides, mmol/L (median [IQR]) | 1.49 [1.05, 2.14] | 1.67 [1.18, 2.40] | 1.04 [0.78, 1.46] | <0.001 | 5.0 |
| Lipoprotein (a), nmol/L (median [IQR]) | 20.00 [9.31, 59.80] | 32.08 [13.60, 64.13] | 67.12 [38.22, 106.97] | <0.001 | 24.0 |
| **Disease status** | | | | | |
| Prevalent T2D (%) | 3623 (2.6) | 380 (9.3) | 181 (4.6) | <0.001 | 0.0 |
| Incident T2D (%) | 4989 (3.6) | 470 (11.6) | 297 (7.6) | <0.001 | 0.0 |
| Incident CHD (%) | 5429 (3.9) | 248 (6.1) | 95 (2.4) | <0.001 | 0.0 |
| Prevalent CHD (%) | 3953 (2.8) | 223 (5.5) | 56 (1.4) | <0.001 | 0.0 |
| Prevalent hypertension (%) | 10840 (7.7) | 465 (11.4) | 465 (11.9) | <0.001 | 0.0 |
| Incident hypertension (%) | 20012 (14.2) | 686 (16.9) | 683 (17.5) | <0.001 | 0.0 |

**Supplementary Table S5. Assessment of detection rate and false positive rate for screening for FH variant carriers using LDL-C cut-off of 4.9mmol/L in European, South Asian and African ancestry groups.** FH+ refers to FH variant carriers.

| Ancestry | **Detection rate using Simon Broome LDL-C >=4.9 mmol/L** | **False positive rate using Simon Broome LDL-C >=4.9 mmol/L** |
|---|---|---|
| European | 37.7% (95% CI: 33.5%; 42.1%) | 8.6% (95% CI: 8.4%; 8.7%) |
| South Asian | 33.3% (95% CI: 16.3%; 56.3%) | 6.3% (95% CI: 5.6%; 7.1%) |
| African | 46.7% (95% CI: 24.8%; 70.0%) | 5.7% (95% CI: 5.0%; 6.4%) |

**Supplementary Table S6. Self-reported statin use in European, South Asian, and African ancestry individuals.** FH+ refers to FH variant carriers.

| Ancestry | Total number of individuals taking statins | Proportion of statin intake per ancestry | Number of FH+ taking statins | Proportion of FH+ taking statins |
|---|---|---|---|---|
| European | 18,405 | 13.1% | 165 | 33.8% |
| South Asian | 826 | 20.3% | 10 | 55.6% |
| African | 488 | 12.5% | 6 | 40.0% |

**MAJOR RESOURCE TABLE**

All data can be accessed via UK Biobank approval system

**Data & Code Availability**

| Description | Source / Repository | Persistent ID / URL |
|---|---|---|
| Genetic data can be access following the UK Biobank approval procedures | UK Biobank Access Management System | https://www.ukbiobank.ac.uk/enable-your-research/apply-for-access |
